# Subthalamic Nucleus Deep Brain Stimulation in Early Stage Parkinson's Disease Is Not Associated with Increased Body Mass Index

**DOI:** 10.1155/2017/7163801

**Published:** 2017-06-06

**Authors:** Sarah H. Millan, Mallory L. Hacker, Maxim Turchan, Anna L. Molinari, Amanda D. Currie, David Charles

**Affiliations:** Department of Neurology, Vanderbilt University Medical Center, 1611 21st Ave S., A-0118 Medical Center North, Nashville, TN 37223-2551, USA

## Abstract

Previous studies suggest that deep brain stimulation of the subthalamic nucleus (STN-DBS) for Parkinson's disease (PD) leads to weight gain. This study analyzes changes in body mass index (BMI) in 29 subjects from a prospective, single-blind trial of DBS in early stage PD (age 50–75, Hoehn & Yahr stage II off medication, treated with antiparkinsonian medications for ≥6 months but <4 years, and without a history of motor fluctuations, dyskinesias, or dementia). Subjects were randomized to DBS plus optimal drug therapy (DBS+ODT; *n* = 15) or ODT (*n* = 14) and followed for 24 months. Weight and height were recorded at baseline and each follow-up visit and used to calculate BMI. BMIs were compared within and between groups using nonparametric *t*-tests. Mean BMI at baseline was 29.7 in the ODT group and 32.3 in the DBS+ODT group (*p* > 0.05). BMI change over two years was not different between the groups (*p* = 0.62, ODT = −0.89; DBS+ODT = −0.17). This study suggests that STN-DBS is not associated with weight gain in subjects with early stage PD. This finding will be tested in an upcoming FDA-approved phase III multicenter, randomized, double-blind, placebo-controlled, pivotal clinical trial evaluating DBS in early stage PD (ClinicalTrials.gov identifier NCT00282152).

## 1. Introduction

Deep brain stimulation of the subthalamic nucleus (STN-DBS) is an FDA-approved adjunctive treatment for Parkinson's disease (PD) when symptoms are no longer adequately controlled by medications. DBS therapy is demonstrated to significantly improve motor symptoms and quality of life for PD patients. Despite its clinical success, isolated studies suggest that STN-DBS is associated with postoperative weight gain and increased body mass index (BMI) [1]. While average weight gain after STN-DBS is reported as a 12.8% increase from preoperative body weight [2], the most significant weight gain typically occurs within the first few months after surgery (8.4% BMI increase [3]), with gradual increases thereafter [1]. These reports of weight gain following STN-DBS are concerning because of the implications for this effective PD therapy leading to additional health complications such as obesity and/or diabetes [4].

STN-DBS is a potent therapy that treats many features of PD that cause weight loss as PD progresses (e.g., dyskinesias and other motor fluctuations and side effects of medical therapy, such as nausea and loss of appetite [5]). For nearly 20 years, DBS has been indicated for advanced stage PD (average disease duration of 10.8 years [6]); this PD patient population has prolonged exposure to the negative effects of the disease progression as well as medication-associated complications leading to considerable weight loss [7]. Therefore, it is not currently clear whether the postoperative weight gain previously reported is due to active STN stimulation or is a consequence of the typical postoperative reduction in medication need and/or the general benefits for PD secondary to DBS therapy.

Vanderbilt University completed a pilot safety and tolerability clinical trial testing STN-DBS in early stage PD (NCT#00282152) [8]. This study offers a unique cohort to evaluate potential postsurgical changes in BMI in early stage PD patients not yet experiencing many of the negative effects related to PD progression. Here, we investigated changes in BMI in the only prospective, randomized clinical trial of STN-DBS in very early stage PD.

## 2. Materials and Methods

Thirty subjects with early stage PD enrolled in the pilot clinical trial. The study was approved by the Vanderbilt University Institutional Review Board (IRB#040797) and the FDA (IDE#G050016). Subjects age 50 to 75 were eligible for enrollment into the study if they were diagnosed with idiopathic PD, treated with medications for more than six months and less than four years, Hoehn & Yahr stage II off medication, and without any history of motor fluctuations or dyskinesias [8–10]. Subjects were excluded if they had any major psychiatric illness, previous brain injury or operative intervention, or contraindications to surgery. A multiphased informed consent process ensured subjects' understanding of the study [11].

Subjects were randomized to receive DBS plus optimal drug therapy (DBS+ODT (*n* = 15) or ODT alone (*n* = 14; one subject dropped out after baseline due to family and career-related circumstances)). Subjects' heights and weights were recorded every six months at each week-long Clinical Research Center (CRC) study visit.

**Figure 1:**
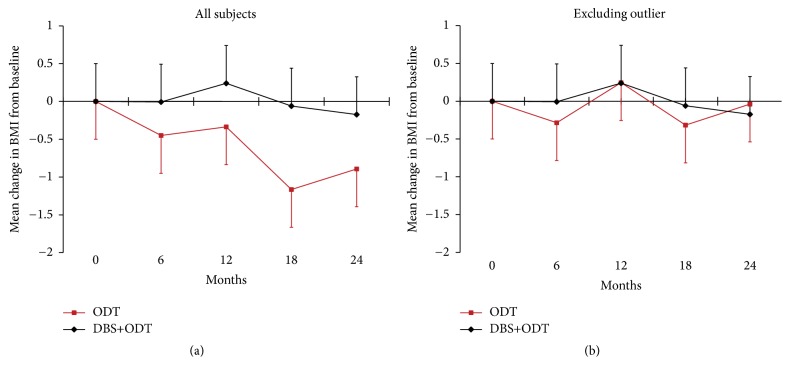
Body mass index change from baseline. (a) Average change in BMI from baseline at 6, 12, 18, and 24 months (± SEM). ODT *n* = 14, DBS+ODT *n* = 15. (b) One subject experienced significant weight loss (BMI decreased by 12 points from baseline to 24 months) and was excluded from this secondary analysis. ODT, *n* = 13; DBS+ODT, *n* = 15.

BMI was calculated at each visit using the height and weight collected on day one of the week-long antiparkinsonian medication and stimulation washout. Mean BMI for each group at baseline, 6, 12, 18, and 24 months was calculated (Figure 1). All within- and between-group comparisons were carried out with nonparametric* t*-tests, Wilcoxon Signed Rank test, and Mann-Whitney *U* test, respectively. Data are reported as mean ± standard deviation (SD) unless otherwise indicated.

## 3. Results

There was no significant difference in average BMI at baseline between the ODT (29.6 ± 4.2) and DBS+ODT groups (32.3 ± 5.7; Table 1; *p* = 0.25). All but one of the subjects in the pilot trial were overweight or obese at baseline (97%, 28/29 with BMI ≥ 25; Table 1).

Over the two-year study period, BMI change for the DBS+ODT group was not significant (*p* = 0.63; Figure 1). Although there was a reduction in average BMI in the ODT group over the two-year period, it was not a significant change from baseline to 24 months (*p* = 0.75). Additionally, the between-group difference in change in BMI score at 24 months was not significant (*p* = 0.62).

There was no BMI change in patients treated with STN-DBS from baseline to the first follow-up visit at 6 months (*p* = 0.65; Figure 1(a)) (prior studies reported the most rapid weight change after the first few months following surgery [4]). One subject in the ODT group experienced a gastrointestinal disorder unrelated to the study, which led to dramatic weight loss over the course of the trial (BMI was reduced by 32.6% from baseline to 24 months). A secondary analysis, conducted with this subject excluded, demonstrated that the slightly lower change in BMI for the ODT group compared to the DBS+ODT group was driven by this subject's extreme weight loss (Figure 1(b)).

## 4. Discussion

These results suggest that STN-DBS is not associated with weight gain in early stage Parkinson's disease. There was minimal change in BMI for the DBS+ODT group over two years (average BMI reduction = −0.17 ± 2.3). Although the BMI for the ODT group decreased slightly over two years (average BMI reduction = −0.89 ± 3.6), this change did not reach significance (*p* = 0.75) and was largely driven by one patient who experienced dramatic weight loss from a gastrointestinal disorder unrelated to the study (Figure 1(b)). These findings suggest that weight gain previously observed in advanced PD patients [1] may not be due to STN stimulation but instead may result from the magnitude of symptom improvement that DBS provides in patients with a more advanced stage of PD.

It is well known that many PD patients experience weight loss with disease progression, and reduced BMI is correlated with increased disease severity [12]. There are many features of advanced PD that likely contribute to weight loss, including increased muscle rigidity, levodopa-induced dyskinesia with increased energy expenditure, and/or depression [5, 7]. Therefore, the great degree of improvement that advanced PD patients experience after STN-DBS therapy more likely explains the weight gain observed in previous isolated studies.

Here, we analyzed an early stage cohort not yet suffering from disabling features of PD that can lead to weight loss, and there was no significant change in BMI after STN-DBS for early stage PD patients. Because this study was open-label, it is possible that BMI changes were influenced by the subjects' awareness of their treatment allocation. Limitations for this study also include the study's small sample size and gender imbalance. It is also important to note that a majority of subjects were overweight or obese at baseline (28/29, Table 1).

Despite its superior clinical benefit over medications alone, one of the* perceived drawbacks* of STN-DBS therapy in advanced PD is its stimulation-associated weight gain [1]. This weight gain is likely due to a variety of factors including postoperative decreased energy expenditure [1] and dosage reduction in PD medications [2]. Since symptoms are typically mild in early stage PD, difference in pre- and postoperative energy expenditure is not expected to change as much as with advanced stage PD.

## 5. Conclusion

These results suggest that STN-DBS does not cause increased BMI in early stage PD. More study is needed to confirm these findings and the FDA has approved a phase III multicenter, randomized, double-blind, placebo-controlled, pivotal clinical trial evaluating DBS in early stage PD.

## Figures and Tables

**Table 1:** Characteristics at baseline^a^.

Characteristic	ODT (*n* = 14)	DBS + ODT (*n* = 15)
Gender		
Male	12	14
Female	2	1
Age (years)		
Mean	60 ± 7.0	60 ± 6.8
Range	51–69	52–74
Baseline medicine use		
Mean duration (years)	2.1 ± 1.1	2.2 ± 1.4
Mean L-dopa equivalents (mg/day)	569 ± 389	451 ± 304
BMI category		
Healthy (18.5 < BMI ≤ 24.9)	1	0
Overweight (24.9 < BMI < 30)	7	7
Obese (BMI ≥ 30)	6	8
BMI (kg/m^2^)	29.7 ± 4.3	32.3 ± 5.7

^a^Modified Table  1 from [10]. Mean ± SD.
